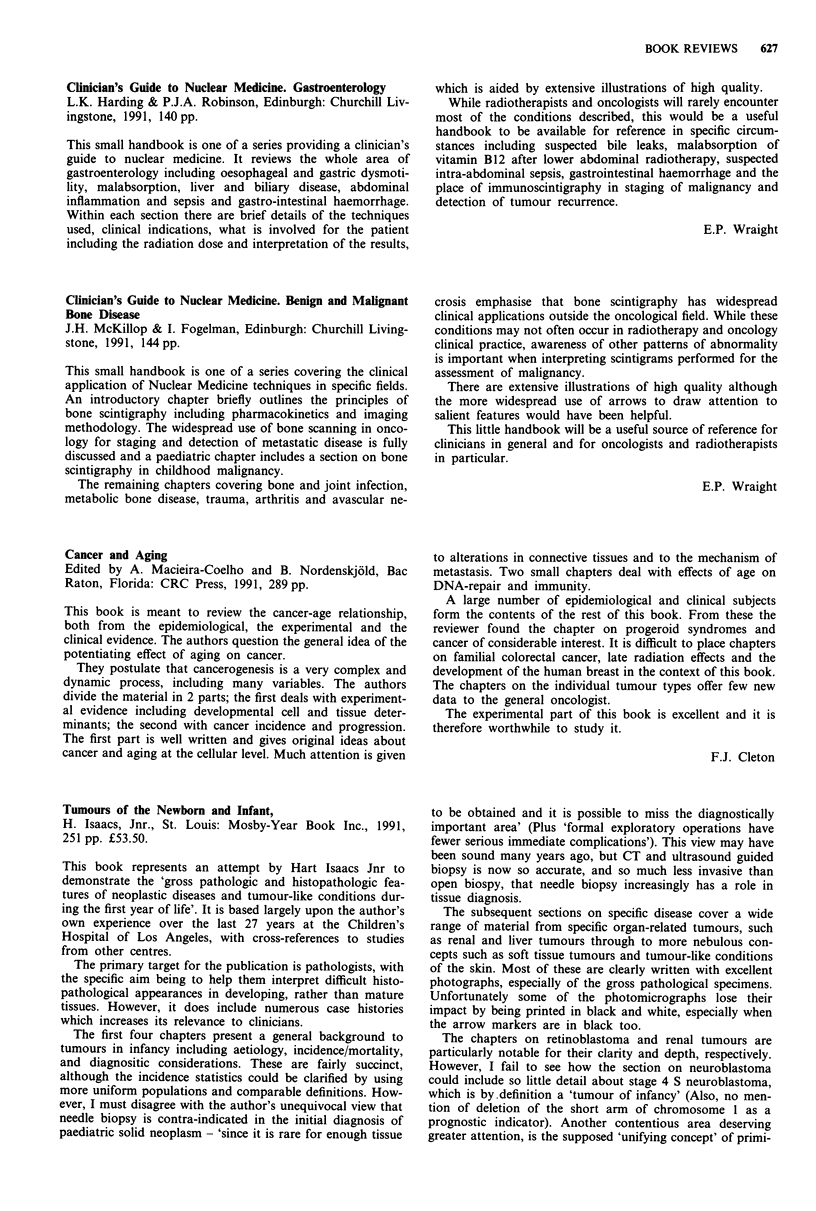# Clinician's Guide to Nuclear Medicine. Gastroenterology

**Published:** 1992-04

**Authors:** E.P. Wraight


**Clinician's Guide to Nuclear Medicine. Gastroenterology**
L.K. Harding & P.J.A. Robinson, Edinburgh: Churchill Livingstone, 1991, 140 pp.

This small handbook is one of a series providing a clinician's guide to nuclear medicine. It reviews the whole area of gastroenterology including oesophageal and gastric dysmotility, malabsorption, liver and biliary disease, abdominal inflammation and sepsis and gastro-intestinal haemorrhage. Within each section there are brief details of the techniques used, clinical indications, what is involved for the patient including the radiation dose and interpretation of the results, which is aided by extensive illustrations of high quality.

While radiotherapists and oncologists will rarely encounter most of the conditions described, this would be a useful handbook to be available for reference in specific circumstances including suspected bile leaks, malabsorption of vitamin B12 after lower abdominal radiotherapy, suspected intra-abdominal sepsis, gastrointestinal haemorrhage and the place of immunoscintigraphy in staging of malignancy and detection of tumour recurrence.

E.P. Wraight